# Etoricoxib as a treatment of choice for patients with *SLCO2A1* mutation exhibiting autosomal recessive primary hypertrophic osteoarthropathy: A case report

**DOI:** 10.3389/fgene.2022.1053999

**Published:** 2022-12-13

**Authors:** Areej Albawa'neh, Mariam Ghareeb Al Mansoori, Sehriban Diab, Fatma Al Jasmi, Nadia Akawi

**Affiliations:** ^1^ Department of Genetics and Genomics, College of Medicine and Health Sciences, United Arab Emirates University, Al-Ain, United Arab Emirates; ^2^ Sheikh Shakhbout Medical City, Abu Dhabi, United Arab Emirates; ^3^ Department of Pediatrics, Tawam Hospital, Al Ain, United Arab Emirates; ^4^ Division of Cardiovascular Medicine, University of Oxford, Oxford, United Kingdom

**Keywords:** autosomal recessive primary hypertrophic osteoarthropathy, *SLCO2A1* gene, etoricoxib, PGE2, digital clubbing

## Abstract

We reported a 22-year-old Emirati male with autosomal recessive primary hypertrophic osteoarthropathy caused by a possibly pathogenic homozygous non-synonymous variant in the *SLCO2A1* gene (NM_005630.3: c.289C>T, p. Arg97Cys) presenting with joint swelling, forehead furrowing, and significant clubbing in all fingers and toes. Currently, no standard treatments are approved for this disease; medical care is palliative and includes non-steroidal anti-inflammatory drugs, corticosteroids, tamoxifen, retinoids, and risedronate. Colchicine may be helpful for the pain due to subperiosteal new bone formation. Our patient was treated with etoricoxib 60 mg once daily and showed a significant clinical improvement at the 6-month mark that was reversed upon the withdrawal of this medication. This case report highlights the importance of placing etoricoxib among first-line therapy recommendations for cases with confirmed primary hypertrophic osteoarthropathy diagnosis. To the best of our knowledge, this is the only case of primary hypertrophic osteoarthropathy from the Middle Eastern population of Arab ethnicity that has responded to non-steroidal anti-inflammatory drug therapy.

## Introduction

Hypertrophic osteoarthropathy (PHO) is an orphan syndrome characterized by abnormal proliferation of the skin and osseous tissues at the distal parts of the extremities. The main clinical features are a peculiar bulbous deformity of the tips of the digits conventionally described as “clubbing,” periosteal proliferation of the tubular bones, and synovial effusions (Martínez-Lavín, 2020). PHO is classified as either primary or secondary, as shown in the following paragraphs. Secondary PHO is more common and always associated with a primary cause (such as cardiac or pulmonary disease), while the primary form of PHO is a rare genetic disease and is further classified based on the range of tissues involved and divided into three subtypes: 1) the complete form, presenting with pachydermia, digital clubbing, and periostosis; 2) the fruste form, with pachydermia and minimal skeletal changes; and 3) the incomplete form, with periostosis and no pachydermia (Zhang et al., 2013).

PHO is characterized by digital clubbing, which is present in almost all cases, arthralgia, or arthritis, commonly affecting the knee, the ankle, and the wrist present in about 20–40% of cases, and periostosis of long bones (Zhang et al., 2013). There is gradual thickening and furrowing of the skin on the face and scalp, resulting in prominent forehead skin folds (cutis verticis gyrata), seborrhea, and blephartosis due to sebaceous gland hypertrophy, and hyperhidrosis due to hypertrophy of sweat glands (Zhang et al., 2013). The precise incidence and prevalence of PHO is still unknown (Zhang et al., 2013). The age of the disease onset has a bimodal distribution, peaking during the first year of life and at puberty. Males are affected predominantly more than females with a ratio of 9:1 (Diggle et al., 2012). Both autosomal recessive (PHOAR) and autosomal dominant (PHOAD) forms of PHO have been described in the literature (Li et al., 2017). Genetic studies have identified the impaired prostaglandin (PGE2) metabolism as the leading cause of PHO, specifically due to biallelic mutations in the *HPGD* gene (MIM*606188) encoding 15-hydroxyprostaglandin dehydrogenase (15-PGDH) or the *SLCO2A1* gene (MIM*601460) encoding the prostaglandin transporter (PGT) (Li et al., 2017). Monoallelic mutations in *SLCO2A1* were also reported to cause PHOAD with incomplete penetrance in heterozygous carriers.

Here, we present the first Emirati patient with a confirmed diagnosis of PHOAR who was treated successfully with etoricoxib for 6 months and showed clinical improvement in symptoms. This report adds more evidence to the existing literature of using etoricoxib as a treatment of choice for PHO disease in different ethnicities.

## Case presentation

**FIGURE 1:**
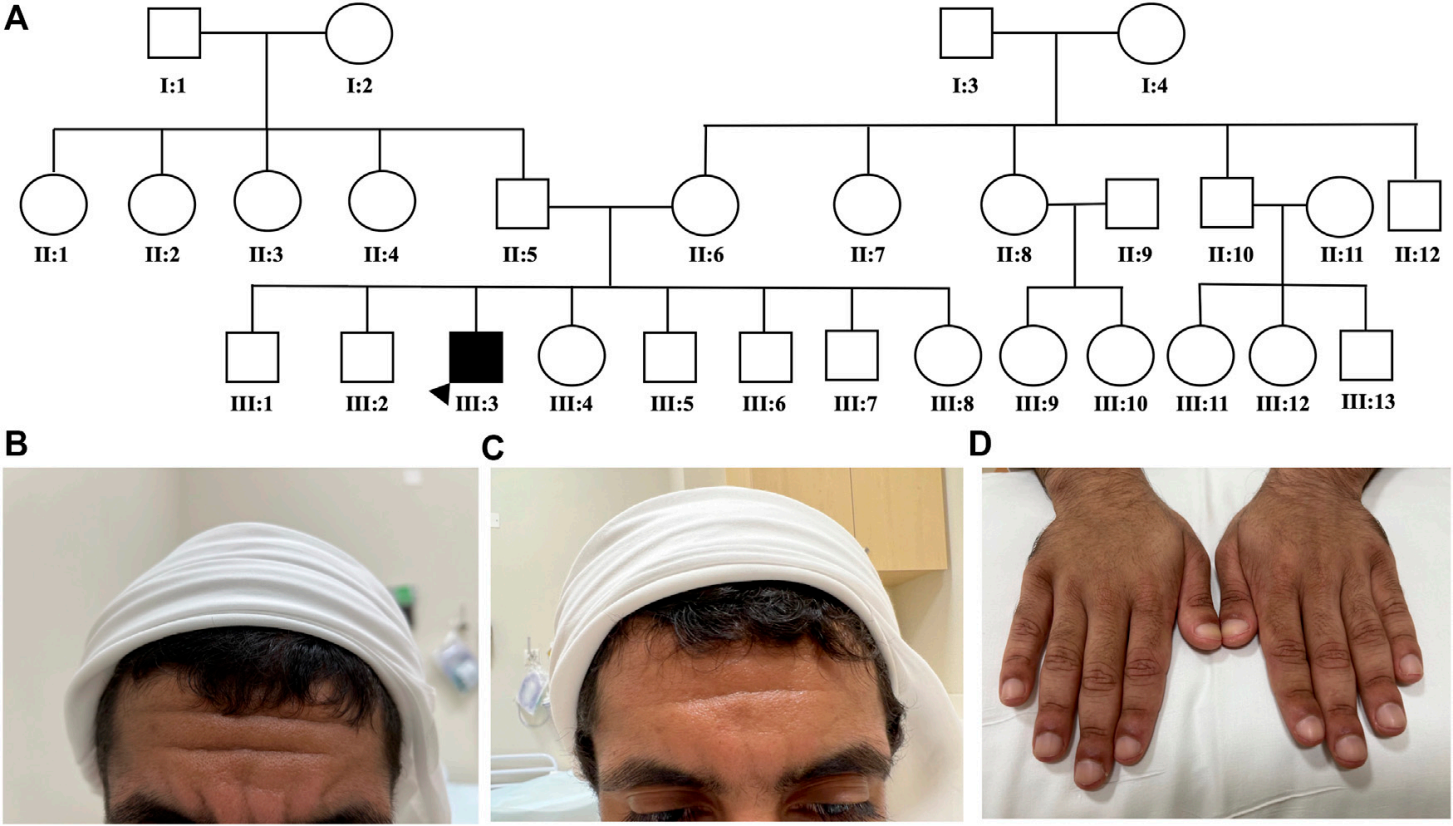
Patients’ family pedigree and characteristics. Patient’s family pedigree showing a negative history of similar conditions **(A)**. Severity of patient’s forehead furrowing is shown before **(B)**, and after **(C)** etoricoxib treatment. A significant improvement in digital clubbing after etoricoxib treatment was observed **(D)**.

A 22-year-old male presented complaints of symmetrical pain in his wrists, hand proximal interphalangeal (PIP) joints, ankles, and feet. This was associated with joint swelling, furrowing of the forehead, and significant clubbing in all fingers and toes. The patient denies any family history of similar symptoms or parent relatedness (Figure 1A). Immunological work-up was negative including the anti-nuclear antibody, rheumatoid factor, anticyclic citrullinated peptide, HLA-B27, and a double-stranded DNA. The C-reactive protein was 15.44 mg/L. Magnetic resonance imaging (MRI) of the right ankle and foot showed an ankle effusion with a healing sprain of the posterior inferior tibiofibular ligament. There were effusions noted in the calcaneocuboid, naviculocuneiform, first tarsometatarsal (TMT), first metatarsophalangeal (MTP) joints, and the second–fifth distal interphalangeal (IP) joints and associated non-specific multifocal marrow edema. The subcortical edema and hyperostosis with mild periostitis of the distal tibia and fibula were concerning for an underlying metabolic bone problem.

The initial working diagnosis was seronegative rheumatoid arthritis, and the patient was treated with intramuscular Kenalog injection (triamcinolone acetonide, 40 mg/ml). However, the patient denied any improvement of his joint pain or swelling and was non-compliant to the methotrexate 12.5-mg tablets weekly that he was commenced on. The patient was seen by several physicians. Two years later, the patient was re-referred to rheumatology to assess for possible inflammatory arthritis after presenting with ongoing arthralgias. Autoimmune serology was negative with a mildly elevated erythrocyte sedimentation rate of 29 and a C-reactive protein of 19.13 mg/L. On musculoskeletal examination, the tender and swollen joint counts were 4/28 (bilateral ankles and knees). An ultrasound of bilateral ankles and knees showed no active synovitis or erosions. Clubbing with red periungual discoloration on all the patient’s fingers and toes was noted. A dermatological examination showed peeling on the lateral aspects of the soles of the feet but no evidence of psoriasis, mucocutaneous or skin ulcers, livedo, malar, or discoid rash. Due to the clinical picture, the lack of synovitis on joint ultrasounds, and the lack of response to IM corticosteroids, the top differential diagnosis was PHO.

## Genetic investigations

Using whole-exome sequencing and unbiased mutational analysis of rare (<1%MAF) pathogenic variants in coding and splicing regions, we detected a homozygous variant on chromosome 3 (GRCh37, Chr3:133692615G>A) that is not found in a homozygous state in gnomAD genomes or exomes, residing in a strongly conserved position. This rare variant is predicted to be a likely pathogenic non-synonymous substitution in exon 3 of the *SLCO2A1* gene (NM_005630.3: c.289C>T, p. Arg97Cys) by 10 pathogenic predictors, namely, BayesDel_addAF, DANN, DEOGEN2, EIGEN, FATHMM-MKL, LIST-S2, M-CAP, MutationAssessor, MutationTaster, and SIFT in VarSome (http://varsome.com). Additionally, this variant was reported as a second hit in a patient with PHO (Li et al., 2017). No pathogenic variant was detected in the *HPGD* gene.

## Treatment and follow-up

**FIGURE 2:**
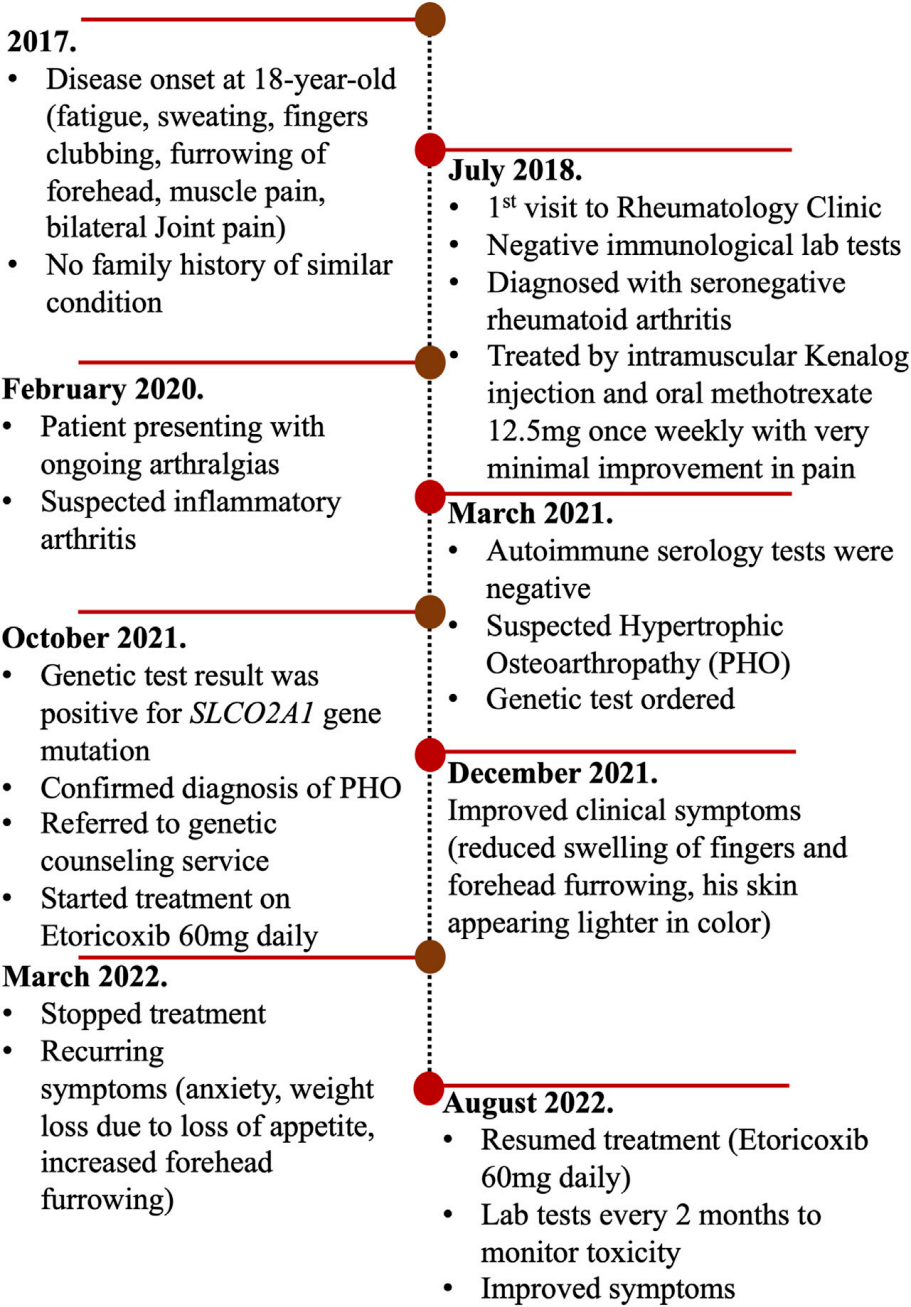
Timeline of the patient’s journey and care presented according to CARE guidelines.

After establishing the diagnosis of primary hypertrophic osteoarthropathy, we reviewed the literature and identified a handful of reports proposing etoricoxib as a treatment for PHO disease (Table 1). We started the patient on a trial of etoricoxib 60 mg once daily, and a clinical improvement was observed within 2 months of treatment on the skin color, sweating, clubbing, weight loss, joint pain, and forehead furrowing, as shown in Figures 1B–D. Treatment with etoricoxib over 6 months was well tolerated, and the patient denied any gastric upset, esophageal reflux symptoms, or diarrhea. The patient and his family were happy with the response to treatment. He also exhibited increased energy levels and self-esteem, and the overall quality of life has improved (Supplementary Table S1). He developed recurring symptoms after discontinuing treatment, which included fatigue, weight loss, clubbing, joint pain, sweating, and furrowing. Six months later, the patient was retreated with the same medication and the same dose (etoricoxib 60 mg once daily) and showed a remarkable improvement in symptoms. To assess treatment safety, regular laboratory tests were conducted every 3 months including a renal function test (eGFR and creatinine), liver enzymes (ALT and AST), and basic metabolic panel. A timeline of the patient’s journey and care was designed to provide a simple and interactive way to review the patient’s medical history using a time-based visualization (Figure 2).

**TABLE 1:** Studies reported treatment of primary hypertrophic osteoarthropathy with etoricoxib.

Author	Dose	Number of patient	Patient’s ethnicity	Variants in *SLCO2A1*	Treatment duration (month)	Clinical outcome	Reference
Li et al.	Etoricoxib 60 mg once a day	41	East Asian	c.941-1G>A P469L, Y357H, R542C, W147X, R542C, N534K, R591X, E60K, G183R, E60K, G554R, W253X G255E, S204L, G460R, P469L, R591X, S432X, Q486K, R603X, R542C, R252X, G369D, Y429X, R280X, E60K, G104R, S432AfsX48, V458F, R97C, G369D, P189S, c.96 + 4A>C, G222R, P219L, Y613X, c.1106-1G>A, N365K, c.861 + 2T>C, Y207X, P457L, and deletion (NC_000003 133692071-133693956)	6	Pachydermia, finger clubbing, and joint swelling significantly improved. Periostosis persisted	Li et al. (2017)
Sun et al.	Etoricoxib 60 mg once a day	1	East Asian	G183R	6	Facial furrows, arthralgia, and joint effusion improved significantly	Sun et al. (2018)
Yuan et al.	Etoricoxib 60 mg once a day	27	East Asian	R603X, G71R, R280X, S70X, F275V, W147X, R542W, L179P, L571P, R591X, Asn545ThrfsX15, R561C, G369D, S481C.1814 + 1C>T, c.470delG, c.830delT, and c.940 + 1G>A	9	Digital clubbing, joint swelling, bone pain, joint pain, pachydermia, and sweating improved	Yuan et al. (2019)
				c.131_134GCC​A>CCT​GT, N534K, c.1795-1798delAAC			
				, F328S, S204L, R542C, G183R, P469C, and c.830delT			
Wang et al.	Etoricoxib 60 mg once a day	2	East Asian	R603X and A286QfsX35	NA	Arthralgia and skin lesions improved. hematochezia and edema persisted, and developed GI complications	Wang et al. (2019)
Xu et al.	Etoricoxib 60 mg once a day	14	East Asian	G554R, G222R, G369D, Q356TfsX77, S432AfsX48, and R603X	3	Improvement in digital clubbing, joint swelling, seborrhea, and acne	Xu et al. (2021)
Prasad et al.	Etoricoxib	1	NA	NA	12	Pain had subsided and motion at the joints had	Prasad et al. (2020)
	Dose NA					improved	
Our patient	Etoricoxib 60 mg once a day	1	Arab	R97C	6	Improvement in skin color, sweating, clubbing, weight, joint pain, and forehead furrowing	This report

NA, not available; GI = gastrointestinal.

## Discussion

**FIGURE 3:**
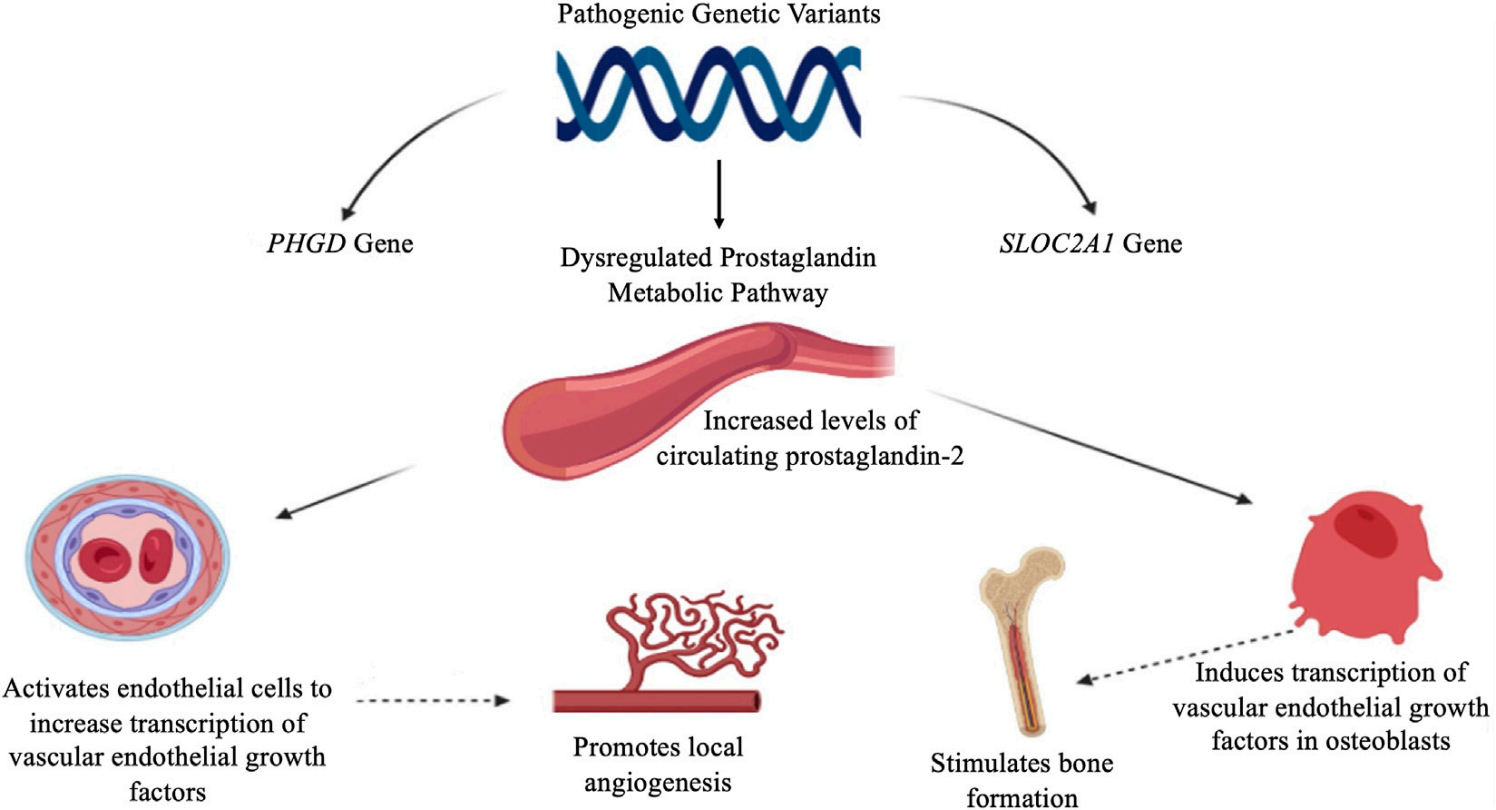
Proposed pathophysiology of primary hypertrophic osteoarthropathy. PGE2 induces the transcription of VEGF in osteoblasts and promotion of endothelial cells, leading to local angiogenesis and a new bone formation. PGE2: prostaglandin E2, VEGF: vascular endothelial growth factor.

We reported a patient exhibiting PHOAR2 (MIM#614441) caused by biallelic mutations in the *SLCO2A1* gene, leading to a defect in the PGE2 metabolism pathway (Zhang et al., 2012). PGE2 is a lipid molecule that promotes hormone-like effects in the body. Derived from arachidonic acid in the cellular membrane, the production of PGE2 is dependent on the cyclooxygenase enzymes 1 and 2 (COX1/2), often elevated in regions of inflammation. Therefore, circulating levels are very low in healthy individuals. PGE2 inactivation, including a cellular uptake by PGT and degradation by 15-PGDH, is important to control bioactive prostaglandin levels at a physiological range. Studies indicated that PHO patients have an elevated circulating PGE2 level and urinary level (Zhang et al., 2012). It has been suggested that increased levels of PGE2 might be responsible for the secondary overexpression of the vascular endothelial growth factor (VEGF) (Martínez-Lavín, 2020). PGE2 induces transcription of the VEGF in osteoblasts and stimulates bone formation, activates endothelial cells that increase transcription of VEGF promoting local angiogenesis, increases capillary permeability, and promotes stimulation and migration of osteoblasts, new bone formation, and edema, all of which account for the characteristic findings in PHO (Zhang et al., 2013), as shown in Figure 3.

Currently, no standard treatment is approved for PHO, and there are no clinical trials except some case reports with varying therapeutic responses (Shakya et al., 2018) (Li et al., 2017). Given the accepted pathophysiology of PGE2-driven overproduction of VEGF, COX-2 inhibitors [non-steroid anti-inflammatory drugs (NSAIDs)], monoclonal anti-VEGF antibodies, steroids, colchicine, tamoxifen, retinoids, and risedronate to alleviate the painful polyarthritis/osteoarthropathy, as well as bisphosphonates (a potent inhibitor of osteoclastic bone resorption), are currently the treatments of choice (Zhang et al., 2013).

There have been various case reports of a symptomatic relief of PHO with the use of bisphosphonates (pamidronate or zoledronic acid) (Jayakar et al., 2011). Steroids can produce hematological improvement but have no effects on the skin or digital clubbing (Giancane et al., 2015). NSAIDs appear to be the best option for improving the musculocutaneous manifestations. Indeed, a systematic review for using NSAIDs among PHO patients concluded that NSAIDs are effective in reducing the arthralgia or arthritis symptoms among PHO patients (Shakya et al., 2018). Etoricoxib is a selective COX-2 inhibitor, approved in Europe for the symptomatic treatment of osteoarthritis, rheumatoid arthritis, ankylosing spondylitis, and acute gouty arthritis, with the potential advantages of convenient once-daily administration and superior gastrointestinal tolerability compared with traditional NSAIDs (Escudero-Contreras et al., 2007). We reviewed the literature and studies that mentioned etoricoxib as treatment for PHO disease and are listed in table 1. Li et al. reported the effect of treatment with etoricoxib on seven patients with PHOAR1 (MIM#259100) and 33 patients with PHOAR2 (Li et al., 2017). A significant reduction in urinary PGE2 levels was observed in the majority of patients during the 6 months of treatment (Li et al., 2017). Moreover, there was a notable improvement in pachydermia, finger clubbing, and joint swelling. However, there was no visible evidence of a positive effect of etoricoxib on periostosis. Xu et al. reviewed 3-month etoricoxib clinical trial results on four patients with PHOAD (MIM#167100) and 33 patients with PHOAR2 and observed an effective response similar to those reported previously by Li et al. (Xu et al., 2021). Additionally, Yuan et al. conducted a single-arm intervention trial to treat 27 PHO patients with etoricoxib 60 mg once daily for a period of 9 months and found that most patients have symptoms improvement during the course of treatment including digital clubbing, joint swelling, bone and joint pain, pachydermia, and sweating (Yuan et al., 2019). However, in that study, one patient discontinued the treatment after 12 months of therapy and developed recurrent symptoms including pachydermia and joint swelling within 1 month.

Consistent with the literature, our patient also responded to etoricoxib treatment for 6 months and documented a notable clinical symptom improvement including the skin, furrowing, diaphoresis, weight loss, and digital clubbing. We also showed the effect of discontinuation of the etoricoxib treatment after the 6-month treatment period on the patient who developed recurrent symptoms. This report adds to the literature as most of the reported patients so far were of East Asian ethnicity, while our Emirati patient is of Arab ethnicity.

Having said that, there are potential side effects for etoricoxib treatment as with all NSAIDs (Reginster et al., 2007), yet the potential gastrointestinal (GI) and cardiovascular risks of treatment with etoricoxib should be weighed against the potential benefits in individual patients. In this context, several clinical trials have been carried out to assess the efficacy and safety of etoricoxib use in chronic conditions, such as osteoarthritis (OA), for example, a study conducted in 617 patients with OA treated with etoricoxib at doses ranging from 30 to 90 mg over 52 weeks demonstrated etoricoxib clinical efficacy in patients with OA and was generally well tolerated (Curtis et al., 2005). Another clinical trial in OA patients treated with etoricoxib 60 mg once daily over a 138-week treatment period demonstrated a long-term clinical efficacy of etoricoxib for the treatment of OA (Reginster et al., 2007).

In summary, etoricoxib is an important treatment option for the management of PHO disease with demonstrated efficacy and safety. Further studies should be conducted to assess the long-term impact of etoricoxib on PHO.

## Data Availability

The datasets presented in this article are not readily available because of federal law restrictions within the UAE. Requests to access the datasets should be directed to the corresponding author.
