# Supplementary material for: Etoricoxib as a treatment of choice for patients with SLCO2A1 mutation exhibiting autosomal recessive primary hypertrophic osteoarthropathy: A case report
Source: Front Genet. 2022 Dec 13;13:1053999. doi: 10.3389/fgene.2022.1053999 (PMC9793897; doi:10.3389/fgene.2022.1053999)
Supplement: Supplementary file 1 [file Table1.docx]

**Supplementary Table 1:** The effect of Etoricoxib treatment on the patient’s quality of life.

| Item | Before Treatment* | After treatment* |
| --- | --- | --- |
| Material comforts home, food, conveniences, financial security | 7 | 7 |
| Health - being physically fit and vigorous | 2 | 4 |
| Relationships with parents, siblings & other relatives- communicating, visiting, helping | 4 | 5 |
| Having and rearing children | 1 | 4 |
| Close relationships with spouse or significant other | 2 | 4 |
| Close friends | 3 | 6 |
| Helping and encouraging others, volunteering, giving advice | 3 | 4 |
| Participating in organizations and public affairs | 2 | 5 |
| Learning- attending school, improving understanding, getting additional knowledge | 3 | 4 |
| Understanding yourself-knowing your assets and limitations - knowing what life is about | 2 | 5 |
| Work - job or in home | 3 | 5 |
| Expressing yourself creatively | 3 | 4 |
| Socializing-meeting other people,  doing things, parties, etc | 1 | 3 |
| Reading, listening to music, or observing entertainment | 7 | 7 |
| Participating in active recreation | 3 | 4 |
| Independence, doing for yourself | 2 | 6 |
| Average | 3 | 4.8 |

*Rate: 1 (Terrible), 2 (Unhappy), 3 (Mostly Dissatisfied), 4 (Mixed), 5 (Mostly satisfied), 6 (Pleased), 7 (Delighted)
